# Atrial fibrillation detected after ischemic stroke (AFDAS) diagnosed by short-term monitoring: the importance of frontal hypoperfusion

**DOI:** 10.1007/s11239-026-03267-7

**Published:** 2026-03-19

**Authors:** Gabriele Prandin, Giovanni Furlanis, Laura Mancinelli, Emanuele Vincis, Magda Quagliotto, Edoardo Ricci, Michele Malesani, Gianpiero Farina, Luigi Cattaruzza, Paola Caruso, Marcello Naccarato, Maja Ukmar, Paolo Manganotti

**Affiliations:** 1 Clinical Unit of Neurology, Department of Medicine, Surgery and Health Sciences, University Hospital and Health Services of Trieste, ASUGI, University of Trieste, Strada di Fiume 447, Trieste, 34149 Italy; 2 Radiology Unit, Department of Medicine, Surgery and Health Sciences, University Hospital and Health Services of Trieste - ASUGI, University of Trieste, Trieste, Italy

**Keywords:** Stroke, CT perfusion, Atrial fibrillation

## Abstract

Atrial fibrillation detected after stroke (AFDAS) may be either newly emergent or previously undiagnosed, with potential neurogenic origin. The ability to predict AFDAS through early imaging remains limited. This study investigates whether hyperacute CT perfusion imaging can identify patients at higher risk for AFDAS. We retrospectively analyzed data of patients with acute ischemic stroke admitted to the University Hospital of Trieste between January 2019 and March 2020. Patients with known AF, hemorrhagic stroke, or TIA were excluded. Lesion volumes and locations were assessed using perfusion and follow-up CT imaging. Among the 242 patients included, AFDAS was more frequently identified in those of older age, female sex, with higher NT-proBNP levels, and worse functional outcomes. Frontal-parietal-insular hypoperfusion was more prevalent in patients with AFDAS. After multiple adjustments, frontal lobe hypoperfusion remained independently associated with AFDAS (OR 2.836, 95% CI 1.406–5.719, *p* = 0.004), while no significant associations were found with final infarct volume or location on follow-up imaging. This finding was confirmed in a sub-analysis restricted to non-lacunar strokes (OR 2.286, 95% CI 1.068–4.894, *p* = 0.033). NT-proBNP levels, non-current smoking status, and frontal hypoperfusion on hyperacute CT perfusion imaging may predict AFDAS, suggesting that early central autonomic dysregulation, rather than infarcted tissue, plays a role in the pathogenesis of atrial fibrillation. These findings support the integration of perfusion imaging and cardiac biomarkers into AFDAS risk stratification models; however, validation through prospective multicenter studies is warranted.

**Graphical Abstract:**

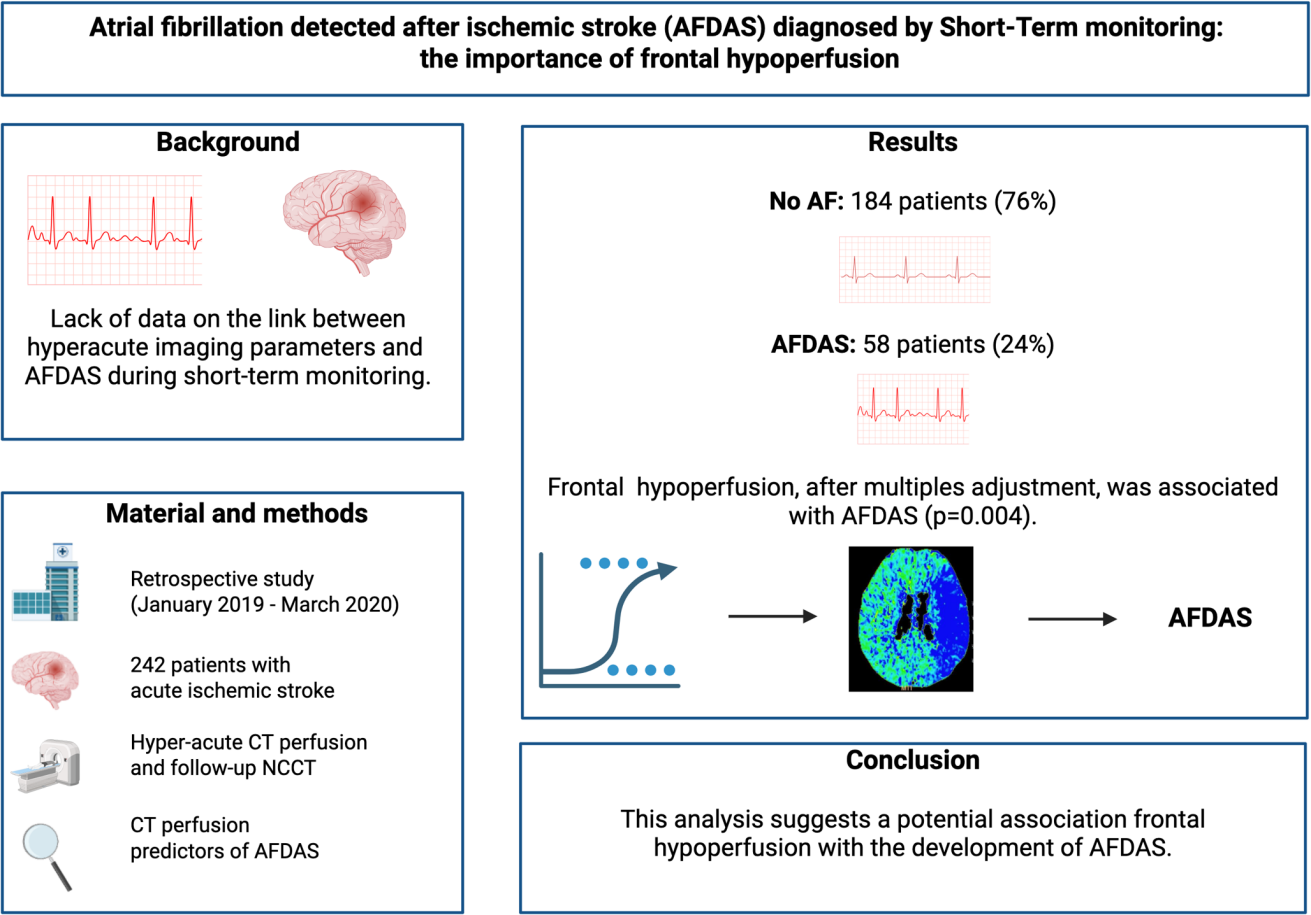

**Supplementary Information:**

The online version contains supplementary material available at 10.1007/s11239-026-03267-7.

## Introduction

Among patients who experience an ischemic stroke, approximately 20% have pre-existing atrial fibrillation (AF), and up to 24% of those without a prior AF diagnosis may be newly diagnosed with AF detected after the stroke (AFDAS) [1]. After completing the diagnostic work-up for ischemic stroke, heart rhythm findings can be categorized into three main groups: patients with previously known AF, patients with AFDAS, and patients in normal sinus rhythm. However, among those with AFDAS, some likely had undiagnosed AF prior to the stroke, while in others, it represents a new-onset arrhythmia. The term AFDAS encompasses two underlying etiologies: cardiogenic and neurogenic [2]. Cardiogenic post-stroke atrial fibrillation (AF) occurs commonly in patients with pre-existing structural heart disease, suggesting that the arrhythmia may have been present prior to the stroke but remained undetected. Conversely, neurogenic AF is thought to result directly from the stroke, potentially triggered by stroke-induced autonomic or neurochemical changes.

Several studies have investigated patient characteristics that may enhance the effectiveness of intensive cardiac monitoring for the detection of AFDAS [3–5]. A large meta-analysis identified multiple risk factors associated with post-stroke AF detection [5], including advanced age, female sex, a history of heart failure, hypertension, ischemic heart disease, elevated National Institutes of Health Stroke Scale (NIHSS) scores, absence of significant carotid or intracranial artery stenosis, non-smoking status, presence of premature atrial contractions, and elevated levels of brain natriuretic peptide. Multiple scoring systems, including the SAFE score, iPAB, and SURF, have been developed to assist clinicians in identifying patients who may benefit from prolonged cardiac monitoring [6–8] for AFDAS detection. However, none of these tools explicitly incorporate perfusion imaging. Of these, only the SAFE score indirectly accounts for cortical involvement based on neuroimaging findings, without specifically referencing perfusion imaging. Recently, a large-scale study suggested a potential predictive role of lesion volume in identifying AFDAS, with a stronger association observed after excluding non-embolic strokes [9]. CT perfusion (CTP) is increasingly becoming a valuable tool in the hyperacute stroke setting. It aids in the diagnosis of stroke mimics [10], patient selection and prognostic assessment in wake-up stroke [11, 12], and evaluation of collateral status in large vessel occlusion [13]. Moreover, CTP provides insight into functional alterations such as crossed cerebellar diaschisis [14]. CTP core volume has also been shown to be more reliable than non-contrast CT (NCCT) in predicting clinical outcomes in patients who achieve endovascular reperfusion within 18 h of symptom onset [15]. However, data on the potential relationship between CTP findings and AFDAS remain scarce.

Early identification of AFDAS is crucial for timely initiation of anticoagulant therapy. Early anticoagulation (within 7 days) after ischemic stroke in patients with atrial fibrillation (either newly diagnosed or previously known) has been found to be safe, reducing mortality even though it does not significantly lower early recurrence rates [16, 17].

The aim of this study is to evaluate which clinical and radiological factors, including hyperacute CT perfusion imaging, can aid in predicting AFDAS.

## Methods

We retrospectively analyzed clinical and radiological data from patients with acute ischemic stroke who were consecutively admitted to the Stroke Unit of the University Hospital of Trieste (Italy) between January 2019 and March 2020. The study was approved by the local Ethics Committee (CEUR – Comitato Etico Regionale FVG, Italy), with approval numbers 115/2018 and 039_2020H. It was conducted in accordance with the principles of the Declaration of Helsinki (1964 and subsequent revisions).

We included patients with ischemic stroke who had acute imaging available comprehensive of hyper-acute CTP and follow up NCCT. Patients admitted within 6 h of known symptom onset or, in the case of wake-up stroke, within 4.5 h of awakening [18, 19]. Patients with hemorrhagic stroke, transient ischemic attacks (TIAs), stroke mimics, or a known history of atrial fibrillation (AF) were excluded.

A standardized stroke work-up was performed for each patient, including assessment of vascular risk factors, electrocardiography, carotid ultrasound, echocardiography, and either Holter ECG or continuous telemetric ECG monitoring.

## Clinical data

Among the clinical data collected, we recorded demographic information (age, sex) and vascular risk factors, including hypertension, diabetes, hypercholesterolemia, history of coronary artery disease, congestive heart failure, current smoking, previous ischemic stroke or intracerebral hemorrhage (ICH), chronic kidney disease (defined as an estimated glomerular filtration rate [eGFR] < 45 ml/min, calculated using the Cockcroft–Gault Eq. [20]), systolic/diastolic and mean blood pressure.

The National Institutes of Health Stroke Scale (NIHSS) score was recorded at both admission and discharge [21]. Functional status was assessed using the modified Rankin Scale (mRS) prior to stroke, at discharge, and at 3-month follow-up. Stroke etiology was classified according to the TOAST (Trial of Org 10172 in Acute Stroke Treatment) criteria [22]. Data on acute management and complications were also collected, including administration of intravenous thrombolysis and/or endovascular thrombectomy, presence of hemorrhagic transformation, and symptomatic intracerebral hemorrhage (sICH), defined according to ECASS II criteria [23].

NT-proBNP (N-terminal pro B-type natriuretic peptide) serum levels were obtained within 48–72 h of admission. The institutional threshold for normal NT-proBNP levels was set at < 300 pg/ml. We also collected echocardiographic data on left atrial enlargement (LAE), assessed by a cardiologist during hospital admission. LAE was considered when its measure exceeds a value of diameter or area personalized for each patient (calculated from age, sex, heart rate and body size).

## AFDAS definition

AFDAS was defined as the evidence of new onset arrhythmia on post-stroke short-term cardiac monitoring (stroke‐unit monitoring and in‐hospital 24–48 h ECG-Holter) up to the patient discharge from the department. Holter monitoring was systematically performed in all patients in whom atrial fibrillation was not documented during standard in-hospital telemetry monitoring. Based on international guidelines, AF was defined as ≥ 30 s of AF or atrial flutter [24, 25]. No-AF was considered when AF was not detected or known before the index stroke. Among the known AF cases, we considered both previously documented AF episodes and AF detected on standard ECG in the emergency department or stroke unit [26].

## CT protocol

At admission, a standardized CT protocol was employed, consisting of non-contrast-enhanced CT (NCCT), single-phase CT angiography (CTA), and CT perfusion (CTP) imaging. Follow-up imaging was performed using NCCT between 22 and 36 h post-admission. All imaging procedures were conducted using a 256-slice CT scanner (Brilliance iCT; Philips Medical Systems, Best, Netherlands). NCCT was acquired at 120 kVp and 400–450 mAs, with a slice thickness of 0.9 mm, reconstructed at 5 mm.

CTP imaging involved intravenous contrast administration at a rate of 4 mL/s, with a total acquisition time of 60 s. Scanning parameters included 80 kVp and 150–200 mAs, utilizing a whole-brain three-dimensional axial acquisition, reconstructed at 5 mm slice thickness. CTP data were processed using the Extended Brilliance Workstation v4.5 (Philips Medical Systems) and a custom-developed MATLAB (MathWorks Inc., Natick, MA) code [27–29].

Perfusion maps—mean transit time (MTT), cerebral blood volume (CBV), and cerebral blood flow (CBF)—were generated using deconvolution-based methods. Time–attenuation curves were modeled via least-squares fitting. MTT was computed through closed-form deconvolution of the voxel-specific time–concentration curve using the arterial input function. CBV was determined as the area under the time–concentration curve for each voxel, and CBF was derived as the ratio of CBV to MTT.

Ischemic lesion volumes on perfusion imaging were calculated from thresholded MTT maps, in which hypoperfused tissue was identified using predefined software-based thresholds (MTT > 7 s or > 145% of the contralateral healthy region, with CBV > 2.0 mL/100 g), as previously described by Wintermark et al. [30]. Volumes were then manually estimated using the ABC/2 method [31–33]. Ischemic lesion volume on 24-h NCCT was quantified using the same approach.

Infarcted areas and perfusion abnormalities, as well as their locations suggestive of ischemia, were qualitatively assessed in the acute setting by a board-certified neuroradiologist (MU). In cases of uncertain imaging interpretation, findings were reviewed and discussed jointly by the radiologist and experienced neurologists from the Stroke Unit (PC, MN, and GF). Some patients did not undergo the advanced imaging protocol because symptom onset occurred outside the reperfusion time windows, or patients had very high pre-stroke disability (mRS), making them unsuitable for thrombolysis or mechanical thrombectomy. Additional factors included contraindications to contrast administration (e.g., allergy or severe renal impairment), hemodynamic or respiratory instability, very mild stroke symptoms (low NIHSS), or extensive infarction already evident on non-contrast CT.

### Statistical analysis

For the statistical analysis, we compared clinical and stroke characteristics between no-AF cohort vs. New-AF. A value of *p* < 0.05 was considered as significant. Univariate and multivariate logistic regression analyses were performed on a subset of selected features (variables that were significantly different between the groups) to identify predictors of AFDAS. Specifically, variables with p-values < 0.05 in the univariate analysis were chosen as candidate predictors for inclusion in the model. Odds ratios (OR) with corresponding 95% confidence intervals (CI) were computed for the logistic regression model.

## Results

**Fig. 1:**
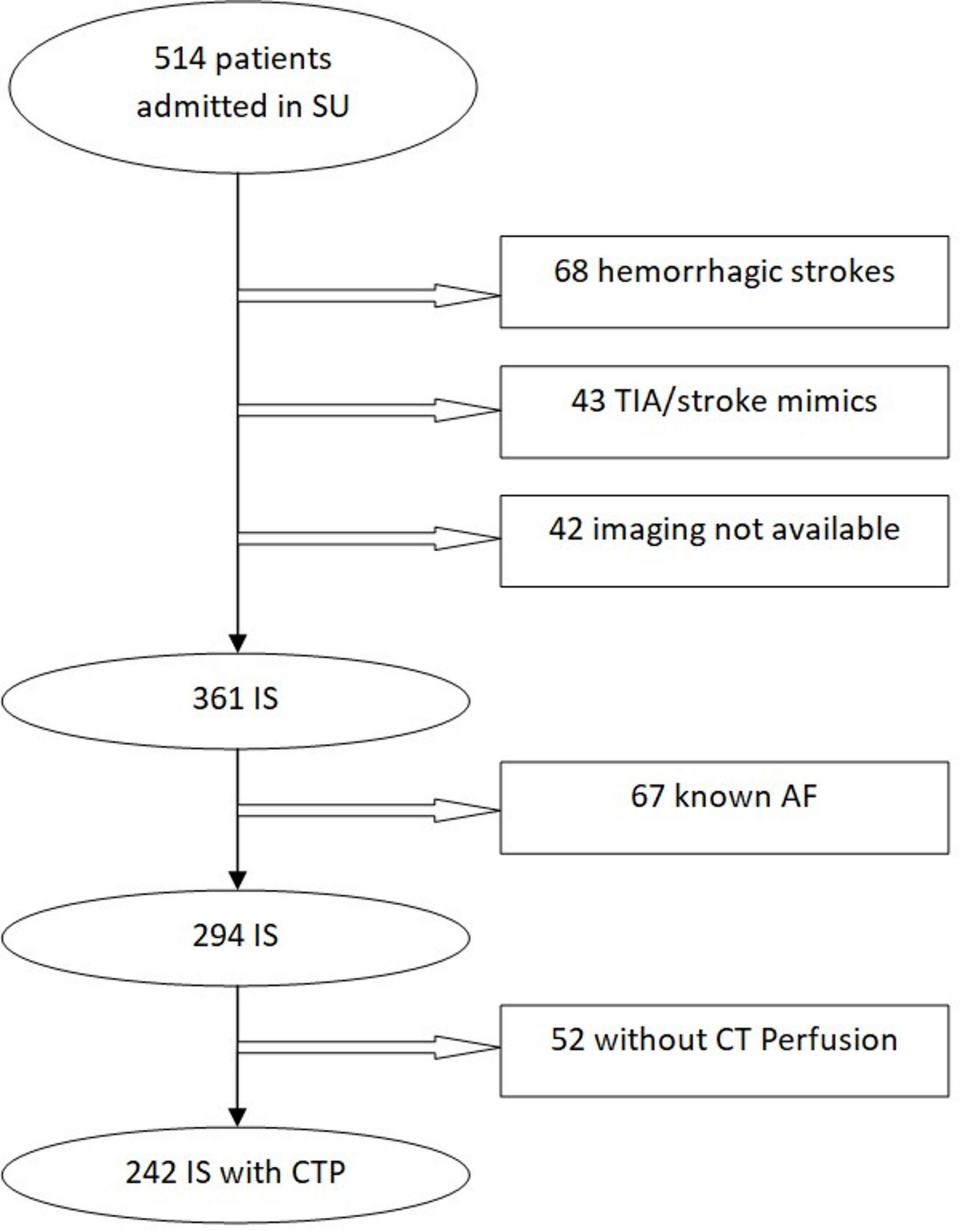
Patients selection

A total of 242 patients were included in the analysis (Fig. 1). The median length of hospital stay was 7 days (interquartile range [IQR], 5–12), and the overall median age was 76 years (IQR, 68–82). The median duration of in-hospital monitoring in the study population was 3 days (IQR, 2–5). 48% of patients (*n* = 116) underwent both SU monitoring and ECG Holter monitoring (23 in the AFDAS group and 93 in the no-AF group, *p* = 0.148). The median Holter duration was 24 h (IQR 23–24) in the AFDAS group and 25 h (IQR 23–44) in the no-AF group, with no significant difference between groups (*p* = 0.989).

Table 1 presents a comparison of demographic characteristics, clinical features, and outcomes between patients without atrial fibrillation (AF) detection and those with AFDAS. The AFDAS cohort was characterized by older age, a higher proportion of female patients, a greater prevalence of congestive heart failure and smoking history, elevated NT-proBNP levels, and worse 90-day functional outcomes as measured by the modified Rankin Scale (mRS). Lacunar etiology was prevalent in No-AF cohort (*n* = 38, 21%) compared to AFDAS (*n* = 3, 5%). Echocardiographic left atrial enlargement differed significantly between groups, being present in 44% (*n* = 54) of the No-AF group and in 70% (*n* = 28) of the AFDAS group (*p* = 0.004). Data on left atrial enlargement assessed by echocardiography were available for 128 patients (70%) in the No-AF cohort and for 40 patients (69%) in the AFDAS cohort.

**Table 1:** Demographic, clinical characteristics and outcomes

	No-AF (*N* = 184)	AFDAS (*N* = 58)	*p*
*Demographics*			
Age, years [median (IQR)]	76 (67–81)	79 (73–85)	**0.002**
Female sex [n, (%)]	86 (47)	41 (71)	**0.001**
*Stroke risk factors and comorbidities*			
Hypertension [n, (%)]	134 (73)	45 (77)	0.471
Diabetes mellitus [n, (%)]	40 (22)	14 (24)	0.702
Hypercholesterolemia [n, (%)]	111 (60)	40 (69)	0.236
Coronary artery disease [n, (%)]	31 (17)	8 (14)	0.581
Congestive heart failure [n, (%)]	13 (7)	12 (21)	**0.003**
NT-proBNP pg/ml [median (IQR)]	389 (147–924)	1730 (673–3561)	**< 0.001**
Current smoking [n, (%)]	46 (25)	3 (5)	**0.001**
Previous ischemic stroke [n, (%)]	19 (10)	4 (7)	0.437
Previous ICH [n, (%)]	1 (1)	0 (0)	1.000
CKD [n, (%)]	25 (14)	8 (14)	0.968
*Stroke characteristics and outcome*			
Admission Systolic BP mmHg [median (IQR)]	144 (133–160)	150 (138–168)	0.275
Admission Diastolic BP mmHg [median (IQR)]	80 (76–90)	81 (70–95)	0.814
Admission Mean BP mmHg [median (IQR)]	104 (96–113)	103 (94–119)	0.961
NIHSS on admission [median (IQR)]	6 (3–13)	10 (4–15)	0.227
NIHSS on discharge [median (IQR)]	1 (0–5)	2 (1–8)	0.155
TOAST etiology [n, (%)]			**< 0.001**
LAA	33 (18)	4 (7)	
CE	9 (5)	45 (78)	
Lacunar	38 (21)	3 (5)	
Other	12 (7)	0 (0)	
Indeterminate/cryptogenic	92 (50)	6 (10)	
Type of treatment [n, (%)]			0.077
No acute treatment	51 (28)	11 (19)	
Thrombolysis	89 (48)	24 (41)	
Mechanical thrombectomy	5 (3)	1 (2)	
Thrombolysis + Mechanical thrombectomy	39 (21)	22 (38)	
90-day mRS 0–2 [n, (%)]	114 (62)	28 (48)	0.065
90-day Mortality [n, (%)]	17 (9)	5 (9)	0.886
Hemorrhagic transformation [n, (%)]	25 (14)	11 (19)	0.316
sICH [n, (%)]	10 (5)	2 (3)	1.000

Bold values are considered as significant (*p* < 0.05)

**Legend**: ICH=intra cerebral haemorrhage; mRS: modified Ranking Scale, NIHSS = National Institutes of Health Stroke Scale., LAA: Large artery atherosclerosis, CE: cardioembolic. Hypertension: Blood Pressure of ≥ 140/90 mmHg at least twice before stroke or already under treatment with antihypertensive drugs; Hypercholesterolemia: total cholesterol ≥ 200 mg/dL or LDL ≥ 160 mg/dL or already on lipid-lowering therapy; Coronary artery disease: myocardial infarction, history of angina or evidence of coronary disease on coronary angiography; CKD= chronic kidney disease defined as an estimated glomerular filtration rate [eGFR] < 45 ml/min; HT: any hemorrhagic transformation; sICH: symptomatic intracerebral haemorrhage according to ECASS II criteria

Table 2 summarizes the radiological characteristics of the two cohorts. The comparison revealed a significantly higher prevalence of frontal-parietal-insular hypoperfusion and larger hypoperfused volumes in the post-stroke AF group. On follow-up NCCT, infarctions involving the frontal lobe was also significantly more frequent in patients with AFDAS.

**Table 2:** Imaging characteristics

	No-AF (*N* = 184)	AFDAS (*N* = 58)	*p*
*CT-Perfusion data*			
Frontal hypoperfusion [n, (%)]	77 (42)	40 (69)	**< 0.001**
Parietal hypoperfusion [n, (%)]	106 (58)	42 (72)	**0.044**
Temporal hypoperfusion [n, (%)]	88 (48)	29 (50)	0.773
Occipital hypoperfusion [n, (%)]	28 (15)	5 (9)	0.202
Insular hypoperfusion [n, (%)]	43 (23)	23 (40)	**0.015**
Basal ganglia/thalamus hypoperfusion [n, (%)]	40 (22)	15 (26)	0.514
Brainstem hypoperfusion [n, (%)]	1 (1)	0 (0)	1.000
Cerebellar hypoperfusion [n, (%)]	6 (3)	3 (5)	0.699
Hypoperfusion volume (ml) [median (IQR)]	31.26 (0.0-71.58)	47.40 (15.60-118.08)	**0.015**
Left Lesion side [n %)]	102 (55)	33 (57)	0.845
*NCCT data*			
Frontal infarction [n, (%)]	68 (37)	31 (53)	**0.026**
Parietal infarction [n, (%)]	69 (38)	29 (50)	0.091
Temporal infarction [n, (%)]	46 (25)	19 (33)	0.245
Occipital infarction [n, (%)]	24 (13)	6 (10)	0.587
Insular infarction [n, (%)]	40 (22)	19 (33)	0.088
Basal ganglia/thalamus infarction [n, (%)]	70 (38)	18 (31)	0.333
Brainstem infarction [n, (%)]	8 (4)	0 (0)	0.204
Cerebellar infarction [n, (%)]	8 (4)	3 (5)	1.000
Final infarction volume (ml) [median (IQR)]	1.98 (0.99–15.37)	3.98 (0.53–17.47)	0.165

Supplementary Table 1 presents detailed left-right comparisons between the AFDAS and no-AF cohorts for the frontal and insular lobes. A significantly higher incidence of hypoperfusion was observed in the left frontal lobe of the AFDAS group compared to the no-AF group (43% vs. 21%, *p* < 0.001), and there was a trend toward greater left insular hypoperfusion in AFDAS patients (24% vs. 14%, *p* = 0.074).

Supplementary Table 2 reports the results of the univariate logistic regression analysis assessing the association between each variable—identified as significantly different in the group comparison—and the presence of AFDAS. Among the variables included, only the hypoperfused volume was not significantly associated with AFDAS.

We conducted multivariate analyses for each stroke location identified on both CTP and follow-up NCCT to adjust for potential confounding factors influencing the development of AFDAS. The results are presented in Table 3.

**Table 3:** Multivariate logistic regression for AFDAS development

	AFDAS §		
	OR	CI 95%	*p* value
*CTP imaging*			
Frontal hypoperfusion	2.836	1.406–5.719	**0.004**
Parietal hypoperfusion	1.536	0.744–3.171	0.245
Insular hypoperfusion	1.718	0.825–3.580	0.148
*NCCT follow-up imaging*			
Frontal infarction	1.786	0.918–3.476	0.088

**§** all variables were adjusted for age, sex, smoke, history of chronic heart failure, NT-proBNP. Together with Frontal lobe hypoperfusion, NT-proBNP (OR 1.002, CI95% 1.001–1.003, *p* = 0.010) and current smoking (OR 0.124, CI95% 0.027–0.555, *p* = 0.006) remain significantly associated with AFDAS

We conducted a sub-analysis limited to embolic strokes, excluding cases of lacunar etiology. Supplementary Tables 3 and 4 provide an overview of the demographic and radiological characteristics of this subgroup, while Supplementary Table 5 presents the results of the univariate and multivariate logistic regression analyses. Consistent with the main analysis, findings in the embolic stroke cohort confirmed that frontal lobe hypoperfusion remained independently associated with post-stroke atrial fibrillation after adjustment for multiple covariates (OR 2.286, CI95% 1.068–4.894; *p* = 0.033).

## Discussion

This study investigated the association between acute perfusion imaging and follow-up non-contrast CT (NCCT) findings with the development of AFDAS. After adjusting for multiple confounders, we found that frontal lobe hypoperfusion was independently associated with AFDAS following the index stroke event. In contrast, none of the NCCT-derived parameters showed a significant association with the predefined outcome.

These findings align with the concept of stroke–heart syndrome (SHS), which encompasses new-onset cardiac dysfunction or the worsening of pre-existing cardiac conditions following an ischemic stroke [34]. The proposed pathophysiology of SHS involves autonomic imbalance—primarily increased sympathetic activity—alongside inflammation and hypothalamic–pituitary–adrenal axis activation [35]. The central autonomic network (CAN) is a key mediator of these processes. Functional MRI studies have identified the frontal lobes and insular cortex as major components of the CAN [36, 37], and non-invasive neuromodulation techniques such as repetitive transcranial magnetic stimulation (rTMS) targeting the prefrontal cortex have been shown to influence cardiac rhythm regulation [38].

Our observation that frontal hypoperfusion, rather than established infarction, was independently associated with AFDAS suggests that early dysfunction of autonomic control—during the hyperacute phase—may be sufficient to impair cardiac rhythm, even when reperfusion ultimately salvages cortical tissue. This is in line with prior findings indicating that early stroke treatment may reduce the risk of cardiac complications by preserving viable cerebral tissue [39, 40].

Notably, our study did not find a significant association with insular cortex involvement, in contrast to previous studies that emphasized its role in autonomic dysregulation and arrhythmogenesis [30, 41]. For instance, a recent MRI-based study involving a large patient cohort reported a significant association between diffusion-weighted imaging (DWI) lesion size and the onset of AFDAS [9]. Differences in imaging modalities (CT perfusion vs. MRI-DWI), sample size, and timing of imaging may partly explain the discrepancy. Conversely, a sub-analysis of the CRYSTAL-AF study found no association between infarct location and the detection of occult AF via long-term cardiac monitoring [42]. Moreover, while a recently published risk model identified cortical infarct location as a predictor of AFDAS [43], it did not specify particular brain regions or include perfusion imaging.

Autonomic imbalance may also exacerbate AF in individuals with subclinical or undiagnosed cardiac vulnerability. Supporting this hypothesis, our study identified NT-proBNP as an independent predictor of AFDAS, reinforcing its role as a marker of early cardiac stress. This finding is consistent with prior evidence showing that elevated NT-proBNP levels at hospital admission are associated with increased risk of AFDAS, with a proposed cut-off of 505 pg/mL [44]. In our cohort, the first quartile NT-proBNP level in the AFDAS group was 688 pg/mL, further supporting this association.

Additionally, systemic inflammation may play a mechanistic role. A recent study demonstrated that elevated C-reactive protein (CRP) levels—both at admission and 24 h post-stroke—were significantly associated with AFDAS onset [45]. As an acute-phase reactant, CRP may reflect inflammation-mediated cardiac remodeling occurring in response to cerebral ischemia [46]. Future studies should consider integrating inflammatory biomarkers alongside neuroimaging to further explore this hypothesis.

Taken together, our findings support a neurogenic mechanism underlying AFDAS, in which acute frontal hypoperfusion may trigger autonomic imbalance and cardiac arrhythmia, independently of established infarction or insular involvement. These results add to the growing body of evidence linking early cerebral dysfunction to post-stroke cardiac events and suggest that perfusion imaging may be a useful tool for identifying patients at risk.

From a clinical practice perspective, it remains debated whether patients with a structurally normal heart and low-burden AFDAS should be anticoagulated. Indeed, the potential embolic risk appears to differ between low-burden AFDAS in patients with a normal heart and high-burden AFDAS in those with underlying structural cardiac abnormalities [47]. In the former scenario, the literature suggests a predominantly neurogenic mechanism—such as autonomic dysfunction and inflammation—rather than a primarily cardiogenic etiology, which is more typical of the latter.

Moreover, “neurogenic” AFDAS seems to be associated with a lower risk of recurrence compared with known atrial fibrillation (KAF), although still higher than in patients without AF [47, 48]. At present, clinical guidelines recommend initiating anticoagulation regardless of the presumed AFDAS subtype. However, in the future, improved patient selection based on structural cardiac characteristics and circulating biomarkers may allow better risk stratification in patients with cryptogenic stroke and help guide decisions regarding anticoagulation in cases of low-burden AFDAS. Further studies are needed to clarify this issue.

This study has several limitations. It is a single-center, retrospective analysis involving a relatively small patient sample. Imaging data were derived from CT modalities (non-contrast CT and CT perfusion), with lesion volumes measured manually, which may introduce inter-rater variability. Moreover Ischemic volumes were estimated using the ABC/2 method, which may be less precise than automated software tools. While we analyzed hypoperfused and ischemic territories rather than the site of arterial occlusion, although these territories are closely related to vascular anatomy, they do not always overlap. We included patients with significant vessel stenosis ipsilateral to the ischemic area, which may have influenced the estimation of MTT volume.

Regarding cardiac monitoring, AFDAS was diagnosed using short-term in-hospital monitoring, and prolonged cardiac monitoring was not performed. Therefore, the possibility of undetected paroxysmal AF or selection bias cannot be excluded but also we cannot provide a more comprehensive assessment of AFDAS beyond the in-hospital setting. Information on AF burden derived from short-term monitoring was not available, and the criteria guiding the use of ECG Holter monitoring may have introduced further bias. Although we excluded patients with a known history of AF, the possibility remains that individuals with undiagnosed subclinical AF prior to stroke onset were inadvertently included in the post-stroke AF group. Existing prediction models for post-stroke AF were developed in cohorts with prolonged cardiac monitoring and may not be directly applicable to our study population, which included only short-term in-hospital monitoring. Furthermore, echocardiographic data were not available for the entire study population.

Given the limited number of AFDAS events, the multivariable model may be prone to overfitting, despite selection of clinically relevant covariates and absence of significant multicollinearity. Additionally, hypoperfused regions and final infarct location are related but capture different pathophysiological aspects. Subgroup analysis restricted to patients with ESUS could provide additional insights, but the number of patients fulfilling strict ESUS criteria in our cohort was small, limiting the feasibility of such analysis.

Finally, data on other cardiac complications—such as acute coronary syndromes, acute heart failure, other arrhythmias, Takotsubo cardiomyopathy—were not collected, as these outcomes were beyond the scope of the present analysis.

Nonetheless, this study reflects real-world clinical practice, incorporating CT-based imaging interpretation and routine NT-proBNP measurement in all patients. Currently, acute CT imaging is more widely available than MRI across stroke centers, and CTP is commonly utilized. These aspects enhance the external validity and generalizability of our findings. However, prospective, multicenter studies with larger sample sizes and more comprehensive data collection are necessary to validate and expand upon these results. Moreover, future studies focusing on cryptogenic stroke may better elucidate the potential role of CT perfusion in predicting AFDAS. 

## Conclusion

This analysis suggests a potential association between elevated NT-proBNP levels, non-current smoking status, and frontal hypoperfusion with the development of post-stroke atrial fibrillation. In contrast, no significant relationship was observed with the location of the final cerebral infarct. Further studies are warranted to validate these findings.

## Supplementary Information

Below is the link to the electronic supplementary material.

Supplementary Material 1

## Data Availability

The data that support the findings of this study are not openly available due to reasons of sensitivity and are available from the corresponding author upon reasonable request.
