# Supplementary material for: Atrial fibrillation detected after ischemic stroke (AFDAS) diagnosed by short-term monitoring: the importance of frontal hypoperfusion
Source: J Thromb Thrombolysis. 2026 Mar 19;59(5):1190–200. doi: 10.1007/s11239-026-03267-7 (PMC13331835; doi:10.1007/s11239-026-03267-7)
Supplement: Supplementary file 1 — Supplementary Material 1 [file 11239_2026_3267_MOESM1_ESM.docx]

**Supplementary Table 1.** Detailed comparisons between right and left side in AFDAS and no-AF cohorts.

|  | **No-AF**  **(N=184)** | **AFDAS**  **(N=58)** | **p** |
| --- | --- | --- | --- |
| **CT-Perfusion data** |  |  |  |
| Left Frontal hypoperfusion [n, (%)] | 39 (21) | 25 (43) | **<0.001** |
| Right Frontal hypoperfusion [n, (%)] | 38 (21) | 15 (26) | 0.403 |
| Left Insular hypoperfusion [n, (%)] | 26 (14) | 14 (24) | 0.074 |
| Right Insular hypoperfusion [n, (%)] | 17 (9) | 9 (16) | 0.178 |
| **NCCT data** |  |  |  |
| Left Frontal infarction [n, (%)] | 40 (22) | 18 (31) | 0.148 |
| Right Frontal infarction [n, (%)] | 28 (15) | 13 (24) | 0.203 |
| Left Insular infarction [n, (%)] | 23 (13) | 11 (19) | 0.217 |
| Right Insular infarction [n, (%)] | 17 (9) | 8 (14) | 0.320 |

**Supplementary Table 2:** univariate binary logistic regression to assess the association of clinical-radiological variables with pre-specified outcome (AFDAS).

|  | UNIVARIATE | |
| --- | --- | --- |
|  | OR (95% CI) | p |
| Female sex | 2.748 (1.456-5.187) | **0.002** |
| Age | 1.045 (1.014-1.076) | **0.003** |
| Smoke | 0.164 (0.049-0.548) | **0.003** |
| Chronic Heart Failure | 3.341 (1.467-8.024) | **0.004** |
| Frontal infarction | 1.959 (1.079-3.556) | **0.027** |
| NT-proBNP | 1.002 (1.001-1.004) | **0.001** |
| Hypoperfused volume | 1.000 (0.999-1.001) | 0.189 |
| Frontal hypoperfusion | 3.088 (1.647-5.790) | **<0.001** |
| Parietal hypoperfusion | 1.932 (1.013-3.895) | **0.046** |
| Insular hypoperfusion | 2.155 (1.151-4.035) | **0.016** |

**Supplementary Table 3:** Demographic, clinical characteristics and outcomes in embolic strokes

|  | **No-AF**  **(N=146)** | **AFDAS**  **(N=55)** | **p** |
| --- | --- | --- | --- |
| **Demographics** |  |  |  |
| Age, years [median (IQR)] | 75 (67-82) | 79 (73-85) | **0.003** |
| Female sex [n, (%)] | 67 (46) | 39 (71) | **0.002** |
| **Stroke risk factors and comorbidities** |  |  |  |
| Hypertension [n, (%)] | 107 (73) | 43 (78) | 0.477 |
| Diabetes mellitus [n, (%)] | 30 (21) | 12 (22) | 0.843 |
| Hypercholesterolemia [n, (%)] | 85 (58) | 39 (71) | 0.099 |
| Coronary artery disease [n, (%)] | 28 (19) | 8 (15) | 0.455 |
| Congestive heart failure [n, (%)] | 11 (8) | 11 (20) | **0.012** |
| Current smoking [n, (%)] | 36 (25) | 3 (6) | **0.002** |
| Previous ischemic stroke [n, (%)] | 17 (12) | 4 (7) | 0.366 |
| Previous ICH [n, (%)] | 0 (0) | 0 (0) | 1.000 |
| CKD [n, (%)] | 20 (14) | 8 (15) | 0.877 |
| NT-proBNP pg/ml [median (IQR)] | 431 (161-1059) | 1733 (673-3584) | **<0.001** |
| **Stroke severity and outcome** |  |  |  |
| NIHSS on admission [median (IQR)] | 7 (3-16) | 10 (4-17) | 0.503 |
| NIHSS on discharge [median (IQR)] | 2 (0-6) | 2 (1-9) | 0.273 |
| 90-day mRS 0-2 [n, (%)] | 88 (60) | 26 (47) | 0.097 |
| 90-day Mortality [n, (%)] | 16 (11) | 5 (9) | 0.699 |
| Acute treatment [n, (%)] | 119 (75) | 45 (82) | 0.959 |
| Hemorrhagic transformation [n, (%)] | 25 (17) | 10 (18) | 0.860 |
| sICH [n, (%)] | 10 (7) | 1 (2) | 0.295 |

**Legend:** ICH=intra cerebral haemorrhage; mRS: modified Ranking Scale, NIHSS = National Institutes of Health Stroke Scale. CKD= chronic kidney disease. sICH: symptomatic intracerebral haemorrhage, LAA: Large artery atherosclerosis, CE: cardioembolic

**Supplementary Table 4.** Imaging characteristics in embolic strokes

|  | **No-AF**  **(N=146)** | **AFDAS**  **(N=55)** | **p** |
| --- | --- | --- | --- |
| **CT-Perfusion data** |  |  |  |
| Frontal hypoperfusion [n, (%)] | 77 (53) | 40 (73) | **0.010** |
| Parietal hypoperfusion [n, (%)] | 106 (73) | 42 (76) | 0.590 |
| Temporal hypoperfusion [n, (%)] | 88 (60) | 29 (53) | 0.333 |
| Occipital hypoperfusion [n, (%)] | 28 (19) | 5 (9) | 0.085 |
| Insular hypoperfusion [n, (%)] | 43 (30) | 23 (42) | 0.096 |
| Basal ganglia/thalamus hypoperfusion [n, (%)] | 35 (24) | 15 (27) | 0.629 |
| Brainstem hypoperfusion [n, (%)] | 1 (1) | 0 (0) | 1.000 |
| Cerebellar hypoperfusion [n, (%)] | 6 (4) | 3 (5) | 0.425 |
| Hypoperfusion volume (ml)[median (IQR)] | 42.12 (11.55-94.22) | 51.61 (20.30-130.41) | 0.287 |
| **NCCT data** |  |  |  |
| Frontal infarction [n, (%)] | 67 (46) | 31 (56) | 0.185 |
| Parietal infarction [n, (%)] | 69 (47) | 29 (53) | 0.489 |
| Temporal infarction [n, (%)] | 46 (32) | 19 (35) | 0.681 |
| Occipital infarction [n, (%)] | 24 (16) | 6 (11) | 0.327 |
| Insular infarction [n, (%)] | 39 (27) | 19 (35) | 0.275 |
| Basal ganglia/thalamus infarction [n, (%)] | 47 (32) | 17 (31) | 0.862 |
| Brainstem infarction [n, (%)] | 2 (1) | 0 (0) | 1.000 |
| Cerebellar infarction [n, (%)] | 6 (4) | 3 (6) | 0.707 |
| Final infarction volume (ml) [median (IQR)] | 3.70 (0.39-20.23) | 4.21 (0.86-21.67) | 0.706 |

**Supplementary Table 5:** univariate and multivariate logistic regression for AFDAS association with clinical-radiological variables in embolic strokes.

|  | **UNIVARIATE** | | **MULTIVARIATE** | |
| --- | --- | --- | --- | --- |
|  | **OR (95% CI)** | **p** | **OR (95% CI)** | **p** |
| **Female sex** | 2.874 (1.475-5.598) | **0.002** | 1.913 (0.903-4.052) | 0.090 |
| **Age** | 1.048 (1.016-1.081) | **0.003** | 1.023 (0.989-1.058) | 0.186 |
| **Smoke** | 0.176 (0.052-0.599) | **0.005** | 0.149 (0.034-0.661) | **0.012** |
| **Chronic Heart Failure** | 3.068 (1.245-7.564) | **0.015** | 1.636 (0.568-4.710) | 0.361 |
| **NT-proBNP** | 1.001 (1.001-1.002) | **0.002** | 1.002 (1.001-1.002) | **0.018** |
| **Frontal hypoperfusion** | 2.390 (1.215-4.700) | **0.012** | 2.286 (1.068-4.894) | **0.033** |
